# Diagnostic stability in adolescents transiting to adult services: exploring the patterns of diagnostic adjustments

**DOI:** 10.1192/j.eurpsy.2023.1550

**Published:** 2023-07-19

**Authors:** N. Baldaquí, C. Crucilla, M. G. Menditto, L. Malvini, A. M. Maltese, M. Percudani, S. Barbera

**Affiliations:** 1 Psychiatry and Psychology Department, Hospital Clínic de Barcelona, Barcelona, Spain; 2 Psychiatry Department, ASST Grande Ospedale Metropolitano Niguarda, Milan, Italy

## Abstract

**Introduction:**

Most mental illness of adult life begin in late adolescence, affecting young people when they require transition to adult services. In <18 years old patients is more difficult to establish a definitive diagnosis, so diagnoses are often unstable and temporary. In young patients, understanding diagnostic stability may help in clarifying the course, developmental changes, and long-term prognoses of psychiatric disorders. Little research has explored the diagnostic adjustments that occur in child and adolescent mental health services, however previous studies reported that moods disorders and schizophrenia showed more stability than other diagnoses.

Understanding diagnostic trajectories is necessary to improve developmental psychopathology, in order to acquire more discrete diagnostic entities, and clinical judgements, regarding risk and prognosis.

**Objectives:**

as the evidence of diagnostic stability from childhood (child and adolescent mental health services) to adulthood (adult services) is limited, the aim of this study is to describe the clinical features in patients from child and adolescent mental health services in transition to adult services and to compare the main diagnosis of these patients made in both services.

**Methods:**

all individuals, between 18 and 25 years old, admitted to our outpatient clinic specialized in prevention, diagnosis and treatment of mental illness in adolescents (ASST Grande Ospedale Metropolitano Niguarda, Milan), referred to our service between 2021 and 2022. Clinical Diagnosis were establish using ICD-10 criteria.

**Results:**

301 new patients were admitted in our outpatients service: 171 in 2021 and 130 in 2022 (until October). The mean age was 21,08. The 30.2% of patients come from child and adolescent mental health services (29,2% in 2021 and 31,5% in 2022). The main diagnosis of these patients were: first reaction to severe stress and adjustment disorders (F43), second specific personality disorders (F60) and thirst eating disorders (F50). The main diagnosis made in our services were: first specific personality disorders (F60), second first reaction to severe stress and adjustment disorders (F43), thirst other anxiety disorders (F41). 56,1% of patients have the same diagnosis in both services and 43.9% have a different diagnosis. There were not differences in sex (60,5% female and 39,5% male). Patients from child and adolescent mental health services were youngers (19,68 vs 21,69), not statistically significant.

**Conclusions:**

Further research is required to understand diagnostic trajectories, especially longitudinal studies in minors during transition period to adult services, in order to find patterns of diagnostic adjustments.

**Disclosure of Interest:**

None Declared

